# Anatomical Distribution of Polyps Is Different for Men and Women With Positive Stool Tests

**DOI:** 10.1002/jgh3.70120

**Published:** 2025-02-21

**Authors:** Joseph C. Anderson, William M. Hisey, Christina M. Robinson, Paul J. Limburg, Bonny L. Kneedler, Lynn F. Butterly

**Affiliations:** ^1^ Geisel School of Medicine at Dartmouth Hanover New Hampshire USA; ^2^ White River Junction VAMC Hartford Vermont USA; ^3^ Department of Medicine Section of Gastroenterology and Hepatology, Dartmouth‐Hitchcock Medical Center Lebanon New Hampshire USA; ^4^ NH Colonoscopy Registry Lebanon New Hampshire USA; ^5^ Exact Sciences Corporation Madison Wisconsin USA

**Keywords:** colon, colonoscopy, stool based screening tests

## Abstract

**Background and Aim:**

Our goal was to inform endoscopist practice by exploring how the odds of advanced neoplasia in the right and left colon differ between men and women with and without prior positive stool tests.

**Methods:**

Our primary outcome was advanced lesions (advanced adenomas, advanced serrated polyps, and/or colorectal cancer) found during colonoscopy. We used logistic regression to compare adjusted outcome odds by colon location (left or right), patient sex, and screening cohort.

**Results:**

Stool‐test+ patients had higher odds for advanced lesions than colonoscopy‐only patients throughout the colon, regardless of sex. While colonoscopy‐only men had significantly higher odds of advanced lesions in the right vs. left colon (OR: 1.87 [1.75–1.99]), the odds of advanced lesions in mt‐sDNA+ and FIT+ men did not differ significantly by colon location. As a result, compared to colonoscopy‐only men, the increase in advanced lesion odds in stool‐test+ men is significantly lower in the right vs. left colon (mt‐sDNA+: OR: 0.63 [0.44–0.90]; FIT+; OR: 0.50 [0.30–0.83]). In stool‐test+ women there was no significant difference in the degree of increase in advanced lesion odds in the right vs. left colon.

**Conclusions:**

Positive stool tests are associated with increased left‐ and right‐sided advanced polyp yield in men and women at colonoscopy. However, stool‐test‐positive men had a significantly higher increase of advanced lesions in the left colon, whereas we found no such differences in women.

**Abbreviations**
AA: Advanced adenoma
BMI: Body mass index
*BMP3*
: Bone morphogenetic protein 3
CI: Confidence interval
CRC: Colorectal cancer
FIT: Fecal immunochemical test
HgB: Hemoglobin
HP: Hyperplastic polyp
IBD: Inflammatory bowel disease
IRB: Institutional review board
*KRAS*
: Kirsten rat sarcoma viral oncogene homolog
mt‐sDNA: Multi‐target stool DNA
*NDRG4*
: N‐Myc downstream‐regulated gene 4
NHCR: New Hampshire Colonoscopy Registry
NSAID: Nonsteroidal anti‐inflammatory drug
OR: Odds ratio
SD: Standard deviation
SP: Serrated polyp
SSP: Sessile serrated polyp
TSA: Traditional serrated adenoma
USMSTF: United States Multi‐Society Task Force on Colorectal Cancer
USPSTF: United States Preventive Services Task Force

## 1 Introduction

The fecal immunochemical test (FIT) and the multi‐target stool DNA (mt‐sDNA) test [1, 2, 3] increase colonoscopy effectiveness by identifying patients with precancerous lesions [4, 5, 6, 7, 8]. Since FIT detects only hemoglobin while mt‐sDNA also identifies DNA mutation and methylation, these two tests have demonstrated differences in serrated and adenomatous polyp detection rates [6, 7, 9, 10]. Evidence from the New Hampshire Colonoscopy Registry (NHCR) suggests that adenoma detection rates can be as high as 63.3% in FIT+ patients and 62.8% in mt‐sDNA+ patients [11]. NHCR data also demonstrate that the sessile serrated polyp prevalence can be as high as 29.2% following a positive mt‐sDNA and 13.5% following FIT+ examinations.

Polyp yield in FIT+ and mt‐sDNA+ patients may differ not only by histology, but also by location in the colon. Because blood from right‐sided lesions may be further metabolized than blood from left‐sided lesions, the HgB antibody used in FIT may be better at detecting left‐sided polyps [12, 13]. In addition to the HgB antibody, mt‐sDNA tests also have methylated markers that can improve detection of serrated neoplasia, commonly located in the right colon [14].

Polyp location may also differ in men and women. Although men may have an overall higher prevalence of conventional adenomas [15], women are more likely to have adenomas in the right colon than in the left [16, 17]. While men are at increased risk for conventional adenomas, women may be at increased risk for serrated polyps, which are relatively more common in the right colon [18]. Evidence on polyp yield and distribution in FIT+ and mt‐sDNA+ men and women could inform endoscopist expectations of the distribution of advanced neoplasia at follow up colonoscopy.

In this analysis, our goal was to inform endoscopist practice by exploring how the odds of advanced neoplasia in the right and left colon differ within and between patients with positive stool tests and patients without any prior test in both men and women, using data from the New Hampshire Colonoscopy Registry (NHCR). In particular, we wanted to determine: (A) whether the odds of finding advanced neoplasia differ between positive‐stool‐test patients and colonoscopy‐only patients on each side of the colon; (B) whether advanced neoplasia odds differ between the left and right colon within each screening cohort; and (C) whether the results are similar for men and women.

Our first hypothesis was that there would be a higher yield of left‐sided polyps, specifically our primary outcome of advanced lesions, in both FIT+ and mt‐sDNA+ men and women compared to colonoscopy‐only patients of the same sex. We also hypothesized that there would be a higher yield of right‐sided lesions, particularly serrated polyps such as sessile serrated polyps, in mt‐sDNA+ women as compared to colonoscopy‐only and FIT+ women. Our main outcome was advanced lesions, which included advanced adenomas, colorectal cancer (CRC), and advanced serrated polyps. However, given the different markers used by the FIT and mt‐sDNA tests, as well as the differences in polyp prevalence by histology in men and women, we also separately evaluated whether there were differences in the odds of finding advanced adenomas and advanced serrated polyps.

## 2 Materials and Methods

### 2.1 Data Collection and Population

The NHCR collects demographic, lifestyle, and family history data from patients undergoing colonoscopies at endoscopy centers across New Hampshire. Data are also collected from the colonoscopies including indication and polyp size and location. Pathology is abstracted by trained staff who match data from pathology to polyp‐level findings.

### 2.2 Study Cohorts

Using an IRB‐approved protocol, Exact Sciences Laboratories LLC (Madison, Wisconsin), provided the NHCR with patient identifiers of all patients with mt‐sDNA+ tests within the NHCR catchment area (New Hampshire, Vermont, Maine, and Massachusetts). We included all patients with a positive stool test (FIT+ or mt‐sDNA+) and a complete colonoscopy with adequate bowel preparation recorded in the NHCR from February 2015 to June 2023. As a reference group, we also included individuals with a screening or surveillance colonoscopy, which was complete with an adequate bowel preparation, without a previous stool test during the same time period. In order to reflect actual practice, in which stool tests are used for patients with no prior colonoscopy findings (screening) as well as with those with a polyp history (surveillance), we included patients in all three cohorts, including our reference group, regardless of prior polyp history. We excluded incomplete exams, exams with poor bowel preparation, exams performed on patients under 50 years old (per the screening guidelines during study period), and those performed for Inflammatory Bowel Disease (IBD), genetic syndromes, serrated polyposis syndrome, or for diagnostic symptoms.

### 2.3 Outcomes

We looked at three different outcomes in both the right and left colon, defining the “right colon” as all segments proximal to the splenic flexure. Our main outcome was *advanced lesions* defined as any CRC, advanced adenoma, or advanced serrated polyp. We also looked separately at *advanced adenomas*, defined as any adenomas > 1 cm, with ≥ 25% villous elements, or with high grade dysplasia, and *advanced serrated polyps*, defined as any Sessile Serrated Polyp (SSP), any Traditional Serrated Adenoma (TSA), or any Hyperplastic Polyp (HP) ≥ 1 cm. We examined outcome prevalence in male and female patients from all three cohorts (mt‐sDNA+, FIT+, and colonoscopy only).

### 2.4 Adjustment Factors

In our adjusted comparisons, we controlled for several other patient‐level characteristics known to affect polyp prevalence and/or potentially related to having a positive stool test: age, Body Mass Index (BMI), smoking status, blood thinner use, previous colonoscopy, and patient risk status for CRC (personal/family history of CRC and/or personal history of polyps).

### 2.5 Statistical and Analytic Approach

We calculated frequencies of men and women with advanced lesions, advanced adenomas, and advanced serrated polyps detected in the right and left colon at colonoscopy in each study cohort. We compared frequencies between the three cohorts using Fisher's exact test separately for men and women.

Next, for each of our three outcomes, we ran logistic regressions to estimate how the odds of polyp findings vary both between the mt‐sDNA+, FIT+, and colonoscopy‐only cohorts and between the right and left sides of the colon for men and women. In order to model these relationships, we created a dataset with two observations for each patient: one for the patient's right colon and one for the left colon. We used a three‐way interaction between patient cohort (mt‐sDNA+ and FIT+ vs. colonoscopy‐only as reference), patient sex (male vs. female as reference), and colon location (right vs. left as reference). We adjusted for patient age (continuous), BMI (continuous), smoking habits (current vs. never/former), blood thinner use, patient CRC risk status (increased vs. average risk), and previous colonoscopy (yes/no). We also performed a sensitivity analysis adjusting only for patient age and CRC risk.

These regression models were used in several ways: (1) to compare adjusted outcome odds in the FIT+ and mt‐sDNA+ cohorts to the colonoscopy‐only reference cohort in both sides of the colon (Table 3); (2) to compare adjusted outcome odds between the right and left colon (reference) for each screening cohort in men and women (Table 4); and (3) to compare the change in outcome odds between the right and the left colon in the mt‐sDNA+/ FIT+ cohorts versus the colonoscopy‐only cohort (Table 5).

## 3 Results

Our sample included 1176 patients with positive mt‐sDNA tests, 584 patients with positive FIT tests, and 68 645 colonoscopy‐only patients (34 284 men and 36 121 women). Demographic and behavioral characteristics are shown in Table 1. For the total sample, 22.5% of mt‐sDNA+ patients, 26.5% of FIT+ patients, and 48.2% of colonoscopy‐only patients were at increased risk of CRC based on a personal/family history of CRC or a personal history of polyps.

**TABLE 1:** Demographic characteristics and patient risk factors in men and women as stratified by screening method.

Men						
	mt‐sDNA+		FIT+		Colonoscopy only	
	*N*/Mean	%/SD	*N*/Mean	%/SD	*N*/Mean	%/SD
Age (continuous)	66.4	8.10	66.2	8.80	62.0	8.11
BMI (continuous)	29.3	6.17	29.3	6.06	29.4	6.60
Smoking status						
Never	164	40.69	108	43.55	15 630	54.73
Former	189	46.90	109	43.95	10 828	37.92
Current	50	12.41	31	12.50	2098	7.35
Increased risk of CRC	123	25.84	84	28.57	16 933	50.53
Patient uses blood thinners						
No	330	84.62	213	86.23	28 034	96.17
Yes	60	15.38	34	13.77	1116	3.83
Patient has had prior colonoscopy						
No	239	50.21	106	36.05	10 527	31.41
Yes	237	49.79	188	63.95	22 987	68.59
Patient has history of neoplastic findings						
No	396	83.19	240	81.63	19 571	58.40
Yes	80	16.81	54	18.37	13 943	41.60
Patient has family history of CRC in first‐degree relative						
No	299	84.94	194	83.26	21 931	78.23
Yes	53	15.06	39	16.74	6103	21.77

Women						
	mt‐sDNA+		FIT+		Colonoscopy only	
	*N*/Mean	%/SD	*N*/Mean	%/SD	*N*/Mean	%/SD
Age (continuous)	66.4	7.90	66.5	8.72	61.4	7.98
BMI (continuous)	28.5	8.09	28.5	8.19	28.3	7.83
Smoking status						
Never	280	49.65	127	50.80	17 420	57.82
Former	214	37.94	102	40.80	10 630	35.29
Current	70	12.41	21	8.40	2076	6.89
Increased risk of CRC	142	20.29	71	24.48	16 148	45.97
Patient uses blood thinners						
No	514	93.97	235	93.63	30 168	98.34
Yes	33	6.03	16	6.37	510	1.66
Patient has had prior colonoscopy						
No	324	46.29	112	38.62	11 297	32.16
Yes	376	53.71	178	61.38	23 834	67.84
Patient has history of neoplastic findings						
No	610	87.27	251	86.55	23 051	65.61
Yes	89	12.73	39	13.45	12 080	34.39
Patient has family history of CRC in first‐degree relative						
No	491	88.15	191	80.93	22 714	75.49
Yes	66	11.85	45	19.07	7373	24.51

### 3.1 Polyp Prevalence and Odds by Location, Study Cohort, and Patient Sex

**FIGURE 1:**
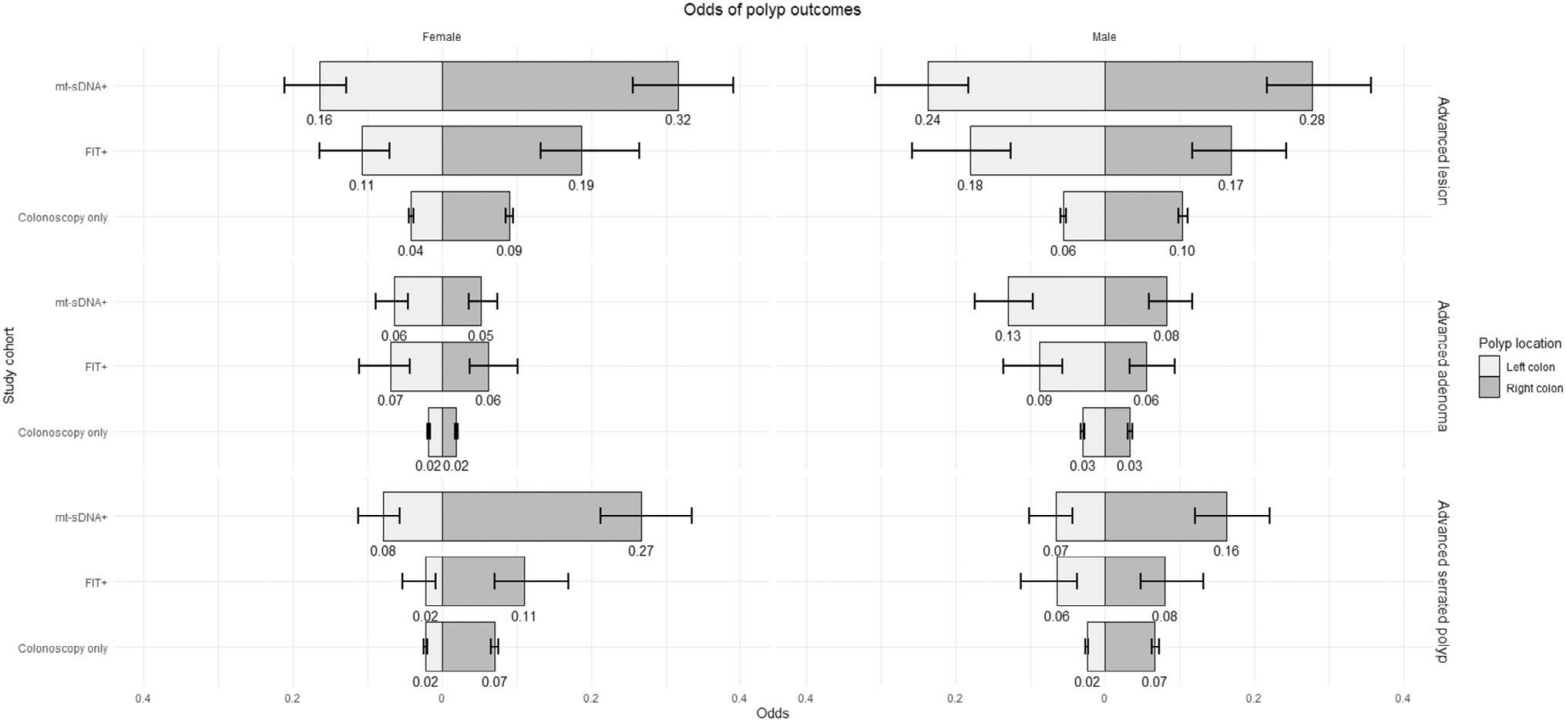
Figure presents the estimated adjusted odds of each polyp outcome in each side of the colon for both men and women, calculated based on our logistic regression results for a 50‐year‐old, non‐smoking, average‐risk, patient with no prior colonoscopy with a BMI of 25 and no current blood thinner use. These results largely reflect the relationships shown in Table 2; both male and female mt‐sDNA+ patients had significantly higher odds of every outcome on both sides of the colon than colonoscopy‐only patients, while FIT+ patients had significantly higher odds for most advanced lesion and advanced adenoma outcomes than colonoscopy‐only patients.

Table 2 presents the polyp prevalence for each patient cohort stratified by patient sex and colon location. Figure 1 presents the estimated adjusted odds of each polyp outcome in each side of the colon for both men and women, calculated based on our logistic regression results for a 50‐year‐old, non‐smoking, average‐risk patient with no prior colonoscopy, a BMI of 25, and no current blood thinner use. The values are available in Table S1. These results largely reflect the relationships shown in Table 2.

**TABLE 2:** Polyp outcome frequency and prevalence for both men and women for all 3 test cohorts.

Left‐sided polyp outcomes in men							
	mt‐sDNA+		FIT+		Colonoscopy only		*p*
	*N*	%	*N*	%	*N*	%	
Any advanced lesion	105	22.0	52	17.7	2017	6.0	< 0.001
Advanced adenoma	75	15.8	35	11.9	1180	3.5	< 0.001
Advanced serrated polyp	32	6.7	17	5.8	858	2.6	< 0.001

Right‐sided polyp outcomes in men							
	mt‐sDNA+		FIT+		Colonoscopy only		*p*
	*N*	%	*N*	%	*N*	%	
Any advanced lesion	120	25.2	48	16.3	3515	10.5	< 0.001
Advanced adenoma	55	11.6	22	7.5	1356	4.1	< 0.001
Advanced serrated polyp	71	14.9	23	7.8	2270	6.8	< 0.001

Left‐sided polyp outcomes in women							
	mt‐sDNA+		FIT+		Colonoscopy only		*p*
	*N*	%	*N*	%	*N*	%	
Any advanced lesion	114	16.3	35	12.1	1522	4.3	< 0.001
Advanced adenoma	57	8.7	25	9.0	712	2.0	< 0.001
Advanced serrated polyp	55	7.9	11	3.8	821	2.3	< 0.001

Right‐sided polyp outcomes in women							
	mt‐sDNA+		FIT+		Colonoscopy only		*p*
	*N*	%	*N*	%	*N*	%	
Any advanced lesion	185	26.4	47	16.2	3129	8.9	< 0.001
Advanced adenoma	47	6.7	21	7.6	766	2.2	< 0.001
Advanced serrated polyp	147	21.0	28	9.7	2429	6.9	< 0.001

*Note:* Advanced adenomas, high grade dysplasia or any adenoma > 1 cm or with ≥ 25% villous elements; Advanced serrated polyp, Sessile Serrated Polyp (SSP), any Traditional Serrated Adenoma (TSA) or Hyperplastic polyps (HP) ≥ 1 cm; Any advanced lesion, CRC, advanced adenoma, or advanced serrated polyp.

### 3.2 Comparisons of Polyp Locations Between the 3 Study Cohorts in Both Men and Women

In Table 3, we present regression results comparing adjusted odds of each outcome in the FIT+ and mt‐sDNA+ cohorts to the colonoscopy‐only reference cohort in both the left and right colon. Among men, mt‐sDNA+ patients had significantly higher odds of all three polyp outcomes in both the right and left colon compared to the colonoscopy‐only cohort. FIT+ patients also had significantly higher odds of advanced lesions in both the right and left colon compared to colonoscopy‐only patients, but had significantly higher odds of advanced adenomas and advanced serrated polyps only in the left colon.

**TABLE 3:** Adjusted odds ratios of polyp findings for positive stool test patients vs. colonoscopy‐only patients by colon location stratified by patient sex.

	Males		Females	
	Left colon	Right colon	Left colon	Right colon
Advanced lesion	OR (95% CI)	OR (95% CI)	OR (95% CI)	OR (95% CI)
Colonoscopy only	REF	REF	REF	REF
FIT+	3.24 (2.26–4.63)	1.62 (1.13–2.34)	2.60 (1.70–3.96)	2.08 (1.47–2.94)
mt‐sDNA+	4.25 (3.28–5.51)	2.67 (2.08–3.42)	3.97 (3.08–5.11)	3.50 (2.84–4.32)

Advanced adenoma	Left colon	Right colon	Left colon	Right colon
Colonoscopy only	REF	REF	REF	REF
FIT+	3.00 (1.94–4.64)	1.65 (0.98–2.77)	3.81 (2.38–6.12)	3.29 (2.01–5.40)
mt‐sDNA+	4.39 (3.26–5.91)	2.45 (1.74–3.45)	3.52 (2.51–4.93)	2.78 (1.93–3.99)

Advanced serrated polyp	Left colon	Right colon	Left colon	Right colon
Colonoscopy only	REF	REF	REF	REF
FIT+	2.70 (1.56–4.68)	1.19 (0.72–1.96)	1.00 (0.41–2.43)	1.56 (1.01–2.41)
mt‐sDNA+	2.73 (1.77–4.21)	2.44 (1.80–3.30)	3.65 (2.58–5.17)	3.80 (3.03–4.76)

Like mt‐sDNA+ men, mt‐sDNA+ women had significantly higher odds of all three polyp outcomes compared to the colonoscopy‐only reference in both sides of the colon. FIT+ women had significantly higher odds of advanced adenomas than the colonoscopy‐only reference cohort on both right and left sides, but only had significantly higher odds of advanced serrated polyps on the right side (OR 1.56, 95% CI 1.01–2.41). Our sensitivity analysis confirmed our Table 3 results (Table S2).

In addition to the results shown in Table 3, we also calculated direct comparisons of right‐sided advanced serrated polyp detection between mt‐sDNA+ and FIT+ patients based on our logistic regression model. Mt‐sDNA+ women had significantly higher odds of right‐sided advanced serrated polyps than FIT+ women (OR: 2.44; 95% CI: 1.50–3.95); similarly, mt‐sDNA+ men had significantly higher odds than FIT+ men (OR: 2.04; 95% CI: 1.15–3.65).

### 3.3 Comparisons Between Right Versus Left Colon Location for Polyp Outcomes Within Each Study Cohort in Men and Women

Table 4 contains comparisons between the odds of polyp outcomes in the right and left colon (reference) for each screening cohort in men and women.

**TABLE 4:** Odds ratios for detecting outcomes in the right versus the left colon within each screening cohort in both men and women.

	Male patients	Female patients
Advanced lesion	Odds ratio (95% CI)	Odds ratio (95% CI)
Colonoscopy only	1.87 (1.75–1.99)	2.18 (2.02–2.34)
FIT+	0.94 (0.56–1.55)	1.74 (1.01–2.99)
mt‐sDNA+	1.17 (0.83–1.67)	1.92 (1.40–2.64)
Advanced adenoma		
Colonoscopy only	1.15 (1.05–1.26)	1.03 (0.91–1.16)
FIT+	0.63 (0.32–1.24)	0.89 (0.45–1.74)
mt‐sDNA+	0.64 (0.41–1.00)	0.81 (0.50–1.31)
Advanced serrated polyp		
Colonoscopy only	2.79 (2.55–3.07)	3.22 (2.93–3.53)
FIT+	1.23 (0.59–2.58)	5.03 (1.87–13.48)
mt‐sDNA+	2.49 (1.49–4.18)	3.35 (2.24–5.00)

In men, we found that colonoscopy‐only patients had significantly higher odds of both advanced lesions and advanced adenomas in the right colon than in the left colon, whereas FIT+ and mt‐sDNA+ patients had no significant differences between sides. Both colonoscopy‐only and mt‐sDNA+ men also had significantly higher odds of advanced serrated polyps in the right colon than in the left, but there was no significant difference between sides in FIT+ patients.

In contrast, among women we found a similar pattern of outcomes in each of the three screening cohorts. All cohorts had significantly higher odds of advanced lesions overall in the right colon than in the left, had no significant difference between sides in the odds of advanced adenomas, and had significantly higher odds of advanced serrated polyps in the right colon than in the left. Our sensitivity analysis confirmed our Table 4 results (Table S3).

### 3.4 Comparing Right Versus Left Colon Location for Polyp Outcomes Between the 3 Test Cohorts for Both Men and Women

Table 5 contains odds ratio ratios (ORR) from the screening cohort/colon location interaction terms in our regressions. These interaction ORRs can be used to compare the change in outcome odds between the right and the left (reference) colon in the mt‐sDNA+/ FIT+ cohorts versus the colonoscopy‐only cohort (reference).

**TABLE 5:** Comparison of advanced outcome odds ratios (colonoscopy‐only reference) between the right and left colon location by patient sex.

	Male patients	Female patients
Advanced lesion	ORR^a^ (95% CI)	ORR^a^ (95% CI)
Colonoscopy only	REF	REF
FIT+	0.50 (0.30–0.83)	0.80 (0.46–1.38)
mt‐sDNA+	0.63 (0.44–0.90)	0.88 (0.64–1.22)
Advanced adenoma		
Colonoscopy only	REF	REF
FIT+	0.55 (0.28–1.08)	0.86 (0.44–1.71)
mt‐sDNA+	0.56 (0.36–0.87)	0.79 (0.48–1.29)
Advanced serrated polyp		
Colonoscopy only	REF	REF
FIT+	0.44 (0.21–0.93)	1.56 (0.58–4.21)
mt‐sDNA+	0.89 (0.53–1.51)	1.04 (0.69–1.57)

^a^ ORR, odds ratio ratio from interaction term between study cohort and location in the colon.

In men, mt‐sDNA+ (ORR: 0.63; 95% CI: 0.44–0.90) and FIT+ (ORR: 0.50; 95% CI: 0.30–0.83) patients both had significantly greater increases in advanced lesion odds in the left colon than in the right colon (Table 5). In women, however, we found no significant difference between the increase in advanced lesion odds in the left colon and in the right colon. Our sensitivity analysis confirmed our Table 5 results (Table S4).

## 4 Discussion

Our goal was to inform endoscopist practice by exploring how the odds of advanced neoplasia in the right and left colon differ within and between patients with positive stool tests and patients without any prior test in both men and women. In this paper, we examined the prevalence and odds of right and left‐sided advanced lesions in men and women with positive stool tests using colonoscopy‐only patients as a reference for expected polyp prevalence.

We found higher odds of right‐ and left‐sided advanced lesions for men and women with positive stool tests than in patients with no prior stool test, even after adjustment. Colonoscopy‐only men had significantly higher odds of advanced lesions in the right colon than in the left, but mt‐sDNA+ and FIT+ men had no significant differences in advanced lesion odds between right and left. Correspondingly, we found stool‐test‐positive men had a significantly lower increase in advanced lesion odds in the right colon than in the left. In FIT+ and mt‐sDNA+ women, however, there was no significant difference in the degree of increased odds of advanced lesions between the right and left colon.

We evaluated two hypotheses for advanced adenoma and advanced serrated polyp prevalence. Our first hypothesis was that stool+ test men and women would have a higher yield of left‐sided advanced adenomas than colonoscopy‐only patients. Prior research suggests that FIT, which has an antibody for human HgB, may have higher sensitivity for lesions in the left side of the colon than the right due to the increased rate of metabolism of HgB from lesions in the proximal (right) colon [12, 13]. After adjusting for important colorectal neoplasia risk factors such as BMI and smoking, we observed a threefold or greater increase in the odds of left‐sided advanced adenomas in both men and women with positive stool tests compared to colonoscopy‐only patients.

While we expected to find an increased left colon advanced adenoma yield for patients with positive stool tests compared to colonoscopy‐only, we also found higher odds of right‐sided advanced adenomas in men with mt‐sDNA+ tests and in women with either positive stool test compared to colonoscopy‐only.

Second, we hypothesized that there would be an increased yield for right‐sided advanced serrated polyps in female patients with mt‐sDNA+ tests compared to FIT+ and colonoscopy‐only patients. After adjusting for covariates, we did find that mt‐sDNA+ women had significantly higher odds of right‐sided advanced serrated polyps compared to FIT+ (OR: 2.44; 95% CI: 1.50–3.95) and colonoscopy‐only (OR: 3.80; 95% CI: 3.03–4.76). Based on this finding in women, we also examined this association in men. Male patients with mt‐sDNA+ tests also had higher odds of right sided advanced serrated polyps as compared to FIT+ and colonoscopy‐only patients.

We also found that mt‐sDNA+ men and women had higher odds of advanced serrated polyps in the left colon compared to the respective colonoscopy‐only cohorts. The increased yield in advanced serrated polyps, regardless of location in the colon, was likely due to the methylated markers present on the stool DNA test. In comparison, there was no increased yield of advanced serrated polyps relative to the colonoscopy‐only cohort in the left colon in FIT+ women.

We also evaluated whether the increases in outcome odds between positive‐stool‐test patients and colonoscopy‐only patients were significantly different between the right and left sides of the colon. Our data demonstrated significantly higher increases in the advanced lesion odds relative to colonoscopy only on the left side than on the right side in FIT+ and mt‐sDNA+ men (Table 5). However, we found no significant differences between the sides in women.

There are few published results examining location of polyps in patients with positive stool tests. One study demonstrated that a high proportion of all polyps in patients with a positive mt‐sDNA test were right‐sided [14]. Some studies examining location of polyps in FIT+ patients observed that lesions detected in these patients were more likely to be left‐sided [12, 13]. Our study is the first to provide data on differences in polyp prevalence by location in men and women with positive stool tests.

Another strength is that our results are from patients who had stool tests in the course of their usual clinical care at multiple centers as opposed to a trial protocol. Data collected from diverse community and academic settings may better represent real world practice and thus may be more generalizable. In addition, the NHCR collects detailed personal data allowing us to adjust for important risk factors such as smoking and BMI.

We acknowledge that there are some limitations in our study. Some of the patients in the sample had neoplasia detected on previous exams. We did not have any information regarding the reason that these patients with prior neoplasia had a stool test as opposed to a surveillance colonoscopy. However, we did adjust for this factor with a variable which accounted for high CRC risk, which included previous neoplasia. In addition, we did not have information on whether trainees assisted in the colonoscopies.

Our findings can help inform endoscopists and referring physicians about expected polyp detection after a positive stool test. In light of the increased yield of serrated polyps found among mt‐sDNA+ patients, particularly in the right colon, it is important to highlight the importance of careful, quality colonoscopy. Because advanced serrated polyps are associated with an increased long‐term risk for CRC [19, 20], an increased ability to detect patients with these serrated polyps is crucial, especially in the right colon, a common site for post‐colonoscopy CRC [21]. Having adequate withdrawal time of 8 min or longer not including time spent on polypectomy or clearing the colon, as well as reintubation of the proximal colon, may help to detect all polyps in patients with positive stool tests.

In summary, we observed that FIT+ and mt‐sDNA+ tests identify men and women with higher odds of significant polyp outcomes in both the right and left colon compared to colonoscopy‐only. However, we found differences between men and women in yield of advanced lesions by location and histology. Endoscopists should be aware of sex‐based differences in the distribution of polyp findings in patients with positive stool tests. Performing quality colonoscopy by employing techniques such as adequate inspection time as well as second look in the proximal colon is essential, perhaps especially in patients with positive stool tests, since those individuals have a known increased risk of important findings.

## Consent

All data collection and study procedures were approved by the Committee for the Protection of Human Subjects at Dartmouth College (CPHS #00015834) in accordance with the Belmont Report and the US Common Rule.

## Conflicts of Interest

The authors declare no conflicts of interest.

## Supporting information


**Table S1.** Odds calculated for a 50‐year‐old, average‐risk, non‐smoking first‐time patient with a BMI of 25 and no blood thinner use.
**Table S2.** Adjusted odds ratios of polyp findings for positive stool test patients vs. colonoscopy‐only patients by colon location stratified by patient sex, adjusted for patient age and risk of colorectal cancer.
**Table S3.** Odds ratios for detecting outcomes in the right versus the left colon within each screening cohort in both men and women, adjusted for patient age and risk of colorectal cancer.
**Table S4.** Comparison of advanced outcome odds ratios (colonoscopy‐only reference) between the right and left colon location by patient sex, adjusted for patient age and risk of colorectal cancer.

## Data Availability

Data were collected by the authors and the non‐protected health information data used in the analyses are available upon reasonable request. Permission to reproduce material from other sources/clinical trial registration: N/A.
