# Supplementary material for: Anatomical Distribution of Polyps Is Different for Men and Women With Positive Stool Tests
Source: JGH Open. 2025 Feb 21;9(2):e70120. doi: 10.1002/jgh3.70120 (PMC11843466; doi:10.1002/jgh3.70120)
Supplement: Supplementary file 1 — Table S1. Odds calculated for a 50‐year‐old, average‐risk, non‐smoking first‐time patient with a BMI of 25 and no blood thinner use. Table S2. Adjusted odds ratios of polyp findings for positive stool test patients vs. colonoscopy‐only patients by colon location stratified by patient sex, adjusted for patient age and risk of colorectal cancer. Table S3. Odds ratios for detecting outcomes in the right versus the left colon within each screening cohort in both men and women, adjusted for patient age and risk of colorectal cancer. Table S4. Comparison of advanced outcome odds ratios (colonoscopy‐only reference) between the right and left colon location by patient sex, adjusted for patient age and risk of colorectal cancer. [file JGH3-9-e70120-s001.docx]

Supplemental Table 1: Odds calculated for a 50-year-old, average-risk, non-smoking first-time patient with a BMI of 25 and no blood thinner use.

| **Male patients** | | |  |
| --- | --- | --- | --- |
| **Advanced lesion** | | |  |
|  |  |  |  |
|  | **Left colon** | **Right colon** |  |
| Colonoscopy only | 0.06 (0.05-0.06) | 0.10 (0.10-0.11) |  |
| FIT+ | 0.18 (0.13-0.26) | 0.17 (0.12-0.24) |  |
| mt-sDNA+ | 0.24 (0.18-0.31) | 0.28 (0.22-0.36) |  |
| **Advanced adenoma** | | |  |
|  |  |  |  |
|  | **Left colon** | **Right colon** |  |
| Colonoscopy only | 0.03 (0.03-0.03) | 0.03 (0.03-0.04) |  |
| FIT+ | 0.09 (0.06-0.14) | 0.06 (0.03-0.09) |  |
| mt-sDNA+ | 0.13 (0.10-0.17) | 0.08 (0.06-0.12) |  |
| **Advanced serrated polyp** | | |  |
|  |  |  |  |
|  | **Left colon** | **Right colon** |  |
| Colonoscopy only | 0.04 (0.04-0.04) | 0.09 (0.09-0.10) |  |
| FIT+ | 0.11 (0.07-0.16) | 0.19 (0.13-0.27) |  |
| mt-sDNA+ | 0.16 (0.13-0.21) | 0.32 (0.26-0.39) |  |
|  | | |  |
| **Female patients** | | |  |
| **Advanced lesion** | | |  |
|  | **Left colon** | **Right colon** |  |
| Colonoscopy only | 0.06 (0.05-0.06) | 0.10 (0.10-0.11) |  |
| FIT+ | 0.18 (0.13-0.26) | 0.17 (0.12-0.24) |  |
| mt-sDNA+ | 0.24 (0.18-0.31) | 0.28 (0.22-0.36) |  |
| **Advanced adenoma** | | |  |
|  | **Left colon** | **Right colon** |  |
| Colonoscopy only | 0.02 (0.02-0.02) | 0.02 (0.02-0.02) |  |
| FIT+ | 0.07 (0.04-0.11) | 0.06 (0.04-0.10) |  |
| mt-sDNA+ | 0.06 (0.05-0.09) | 0.05 (0.04-0.07) |  |
| **Advanced serrated polyp** | | |  |
|  | **Left colon** | **Right colon** |  |
| Colonoscopy only | 0.02 (0.02-0.02) | 0.07 (0.07-0.08) |  |
| FIT+ | 0.02 (0.01-0.05) | 0.11 (0.07-0.17) |  |
| mt-sDNA+ | 0.08 (0.06-0.11) | 0.27 (0.21-0.33) |  |

**Supplementary Table 2 Adjusted odds ratios of polyp findings for positive stool test patients vs. colonoscopy-only patients by colon location stratified by patient sex, adjusted for patient age and risk of colorectal cancer**

|  | **Males** | | **Females** | |
| --- | --- | --- | --- | --- |
|  | **Left colon** | **Right colon** | **Left colon** | **Right colon** |
| **Advanced lesion** | OR (95% CI) | OR (95% CI) | OR (95% CI) | OR (95% CI) |
| Colonoscopy only | REF | REF | REF | REF |
| FIT+ | 3.41 (2.52-4.62) | 1.69 (1.24-2.31) | 3.07 (2.14-4.39) | 2.00 (1.46-2.74) |
| mt-sDNA+ | 4.50 (3.61-5.62) | 2.93 (2.37-3.62) | 4.37 (3.55-5.38) | 3.74 (3.15-4.45) |
|  |  | |  | |
| **Advanced adenoma** | **Left colon** | **Right colon** | **Left colon** | **Right colon** |
| Colonoscopy only | REF | REF | REF | REF |
| FIT+ | 3.55 (2.48-5.08) | 1.84 (1.18-2.85) | 4.31 (2.84-6.54) | 3.31 (2.11-5.19) |
| mt-sDNA+ | 4.91 (3.81-6.34) | 2.97 (2.23-3.96) | 4.07 (3.07-5.40) | 3.07 (2.26-4.17) |
|  |  | |  | |
| **Advanced serrated polyp** | **Left colon** | **Right colon** | **Left colon** | **Right colon** |
| Colonoscopy only | REF | REF | REF | REF |
| FIT+ | 2.47 (1.50-4.05) | 1.23 (0.80-1.89) | 1.75 (0.95-3.20) | 1.53 (1.03-2.26) |
| mt-sDNA+ | 2.91 (2.02-4.20) | 2.56 (1.98-3.31) | 3.80 (2.86-5.06) | 3.82 (3.17-4.61) |

**Supplementary Table 3. Odds ratios for detecting outcomes in the right versus the left colon within each screening cohort in both men and women, adjusted for patient age and risk of colorectal cancer**

|  | **Male patients** | **Female patients** |
| --- | --- | --- |
| **Advanced lesion** | Odds ratio (95% CI) | Odds ratio (95% CI) |
| Colonoscopy only | 1.83 (1.73-1.94) | 2.16 (2.03-2.30) |
| FIT+ | 0.91 (0.59-1.40) | 1.41 (0.88-2.26) |
| mt-sDNA+ | 1.19 (0.88-1.61) | 1.85 (1.42-2.40) |
| **Advanced adenoma** | |  |
| Colonoscopy only | 1.16 (1.07-1.25) | 1.08 (0.97-1.19) |
| FIT+ | 0.60 (0.34-1.05) | 0.83 (0.45-1.51) |
| mt-sDNA+ | 0.70 (0.48-1.01) | 0.81 (0.54-1.21) |
| **Advanced serrated polyp** |  |  |
| Colonoscopy only | 2.77 (2.55-3.00) | 3.10 (2.86-3.37) |
| FIT+ | 1.38 (0.72-2.65) | 2.71 (1.32-5.56) |
| mt-sDNA+ | 2.43 (1.57-3.77) | 3.12 (2.24-4.34) |

**Supplementary Table 4. Comparison of advanced outcome odds ratios (colonoscopy-only reference) between the right and left colon location by patient sex, adjusted for patient age and risk of colorectal cancer**

|  | **Male patients** | **Female patients** |
| --- | --- | --- |
| **Advanced lesion** | **ORR^1^ (95% CI)** | **ORR^1^ (95% CI)** |
| Colonoscopy only | REF | REF |
| FIT+ | 0.50 (0.32-0.77) | 0.65 (0.41-1.05) |
| mt-sDNA+ | 0.65 (0.48-0.88) | 0.86 (0.65-1.12) |
| **Advanced adenoma** | | |
| Colonoscopy only | REF | REF |
| FIT+ | 0.52 (0.29-0.91) | 0.77 (0.42-1.42) |
| mt-sDNA+ | 0.60 (0.41-0.89) | 0.75 (0.50-1.14) |
| **Advanced serrated polyp** | | |
| Colonoscopy only | REF | REF |
| FIT+ | 0.50 (0.26-0.96) | 0.87 (0.42-1.80) |
| mt-sDNA+ | 0.88 (0.56-1.37) | 1.00 (0.72-1.41) |
| 1: ORR: Odds Ratio Ratio from interaction term between study cohort and location in the colon | | |
